# Corrosion Inhibition Coating Based on the Self-Assembled Polydopamine Films and Its Anti-Corrosion Properties

**DOI:** 10.3390/polym14040794

**Published:** 2022-02-18

**Authors:** Shanjian Li, Changwang Guo, Xin Wang, Chong Guan, Gang Chen

**Affiliations:** 1 Shaanxi Province Key Laboratory of Environmental Pollution Control and Reservoir Protection Technology of Oilfields, College of Chemistry and Chemical Engineering, Xi’an Shiyou University, Xi’an 710065, China; gcw_2021@163.com (C.G.); gangchen@xsyu.edu.cn (G.C.); 2 No. 3 Gas Production Plant of Yanchang Gas Field of Shaanxi Yanchang Petroleum Co., Ltd., Yan’an 716000, China; wxin_yc@163.com (X.W.); guanchong088@163.com (C.G.); 3 State Key Laboratory of Petroleum Pollution Control, Xi’an Shiyou University, Xi’an 710065, China

**Keywords:** dopamine, anti-corrosion coating, corrosion rate, oil well produced water, gas well produced water

## Abstract

Metal corrosion is becoming increasingly serious in oil and gas production, and one way to solve this problem is to modify the metal surface. Thus, a corrosion inhibition coating on the N80 steel was constructed via the self-polymerization and assembling of the dopamine. The optimum reaction condition of polydopamine films was determined by the corrosion rate assessment of the films coated N80 steel, which was the reaction at 60 °C and 5 g/L dopamine in the Tris-HCl buffer solution (pH = 8.5) for 1 h. The spectral results confirmed the existence of the polydopamine coating on the surface of N80 steel, and high stability of the coating in the oil well produced water was observed. The anti-corrosion performance of the polydopamine-coated N80 steel confirmed that high temperature accelerated the anti-corrosion effect of the coating, and the corrosion rate of N80 plate in 90 °C oil well produced water was 0.0591 mm·a^−1^, lower than the standard value. The corrosion rates of the polydopamine coated N80, A3 and J55 plates at 90 °C were 0.0541 mm·a^−1^, 0.0498 mm·a^−1^ and 0.0455 mm·a^−1^, respectively. No significant effects of the categories of corrosive medium and steel plate on the performance of the coating were observed.

## 1. Introduction

At present, the water content of the produced fluid in some oil and gas fields is getting higher and higher [1], and it is often accompanied by high-salinity, high-temperature, acid gas impurities [2]. Thus, the anti-corrosion of wellbore, pipelines and related facilities is a continuous task [3,4,5,6,7]. Most oil and gas wellbore and pipeline corrosion inhibitors need to be continuously injected into the fluid for corrosion inhibition, which requires additional work on the part of on-site operators [8,9,10]. One attractive anti-corrosion strategy is to modify the surfaces of wellbore and pipeline to make them have stable corrosion resistance during the production, while reducing the workload.

Inspired by mussel adhesion proteins in the ocean, Messersmith et al. [11,12] found that dopamine can undergo oxidative self-polymerization in a weakly alkaline environment, thereby forming a polydopamine film on the surfaces of various materials. In the presence of dissolved oxygen, dopamine oxidizes and self-polymerizes to generate polydopamine, which can form a polydopamine coating on the surface of materials of any composition and shape. Polydopamine has been applied to coat various materials, including metals (Au, Ag, Pt), oxides (TiO_2_, SiO_2_, Al_2_O_3_, Nb_2_O_5_), semiconductors, ceramics, synthetic polymers (polystyrene PS, polyethylene PE, polydimethylsiloxane PDMS, polyurethane PU), etc. This kind of polydopamine has active groups such as catechol and amino groups, which can further undergo Michael addition reactions and Schiff base reactions with molecules containing amino groups or sulfhydryl groups, or coordination reactions with metal ions, thereby functionally modifying the material. Polydopamine has shown outstanding advantages in the field of surface modification and has been developed by leaps and bounds in recent years. Elmi et al. [13] reported polydopamine coating on 304 stainless steel (304ss), was successfully prepared using a self-assembly process. The inhibitory properties of the coatings were verified by potentiodynamic polarization measurements and electrochemical impedance spectroscopy in sterile seawater. Habibiyan et al. [14] investigated that the prepared GO-PDA and GO-PDA-Zn nanocomposites exhibit different ion-capturing/releasing properties derived from opposite charges developed on them. This property not only declines the permeation of aggressive species into the coating matrix but also facilitates the release of divalent zinc ions or PDA through an on-demand manner when local corrosion initiates at the damaged zone. Bahremand et al. [15] studied the post-modification of Sm-modified plates by polydopamine biopolymers, which were synthesized by the self-polymerization and oxidant induction. EIS results evidenced the significant impact of the post-treatment of the Sm-treated samples by polydopamine (PDA) nanoparticles (NPs) on its corrosion inhibition ability enhancement.

In this study, an anti-corrosion coating based on self-aggregation and -assembled polydopamine was prepared on the surface of N80 steel. After that, the key reaction conditions during the construction of anticorrosive coatings were optimized by static weight loss. In addition, the surface of the coating was characterized by infrared spectroscopy, and the stability of anticorrosive coating in oil well produced water was evaluated. Then, static weight loss method and electrochemical analysis were adopted to study the corrosion inhibition performance of polydopamine coating on steel under different corrosion temperatures, corrosion media (produced water from oil wells, produced water from gas wells, and acid solution) and steel substrates (N80, A3 and J55 steel). It is hoped that the results can provide useful reference for research and application of corrosion inhibition in wellbores and pipelines.

## 2. Materials and Methods

### 2.1. Instruments and Reagents

Instruments: Electronic balance (JEB2002, Jinan Blue Arrow Weighing Technology Co., Ltd., Jinan, China), ultrasonic cleaner (VGT-1620QTD, Wuxi Maggi Ultrasonic Equipment Co., Ltd., Wuxi, China), vacuum drying oven (DZ-2BC II, Hangzhou Instrument Equipment Co., Ltd., Hangzhou, China), electrochemical workstation (CS350, Hangzhou Instrument Equipment Co., Ltd., Hangzhou, China), ultraviolet-visible spectrophotometer (UV-2600, Thermo Nicolet Corporation, WI, USA), infrared spectrometer (Spectrum II, Thermo Nicolet Corporation, WI, USA).

Reagents: dopamine (analytical grade), tris (analytically pure) and concentrated hydrochloric acid (analytically pure) purchased from Tianjin Kemiou Chemical Reagent Co., Ltd. (Tianjin China); sodium acetate (chemically pure) and copper sulfate (analytically pure) from Xi’an Chemical Reagent Factory (Xi’an, China); acetone (analytically pure) and ethanol (analytically pure) from Rionlon Bohua (Tianjin, China) Pharmaceutical Chemical Co., Ltd.; a 30% H_2_O_2_ solution from Sinopharm Group(Shanghai, China); sodium nitrite (analytically pure) from Tianjin Tianli Chemical Reagent Co., Ltd. (Tianjin, China); Sodium hydroxide (analytically pure) from the experimental plant of Shanghai Chemical Technology College (Shanghai, China).

The corrosive media were oil well produced water, gas well produced water, and hydrochloric acid solution (pH = 3), as shown in Table 1.

Three kinds of steel materials were used in the experiment, and their chemical compositions are listed in Table 2.

### 2.2. Coating Preparation and Characterization

#### 2.2.1. Coating Preparation

**Figure 1:**
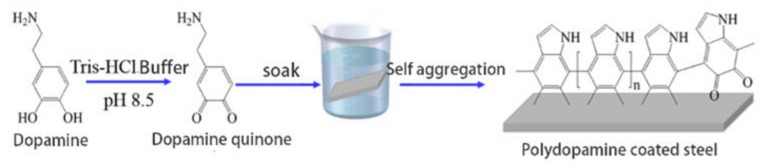
Preparation process of polydopamine coating on steel surface.

First, the concentrated hydrochloric acid was diluted with distilled water to 0.1 mol/L for later use. Tris was quantitatively weighed and dissolved in distilled water, and 0.1 mol/L dilute hydrochloric acid was added to adjust the solution to the specified pH to obtain Tris-HCl buffer. The steel plate was polished with 400#, 800# and 1200# sandpaper in turn, washed with 0.1 mol/L NaOH solution, distilled water and absolute ethanol in an ultrasonic cleaner for 5 min, dried and weighed. After that, dopamine was quantitatively weighed and dissolved in 100 mL of TrIS-HCL buffer solution, which was then added with 0.1 mol/L additives (copper sulfate and anhydrous ethanol), and stirred in dark at a constant speed for 0.5 h. Subsequently, the polished and cleaned steel plate was soaked in the dopamine solution and taken out after standing at 30 °C for 12 h. After washing away its residual liquid with distilled water, the steel plate was dried and weighed for later use. Figure 1 shows the coating preparation process. In an alkaline environment, dopamine is first oxidized to form dopamine-quinone, and then undergoes intramolecular cyclization via Michael addition reaction. The product from intramolecular cyclization undergoes further oxidation and molecular rearrangement reactions to form 5,6-dihydroxyindole, which is easily oxidized to form 5, 6-indolequinone. The two products are prone to branching reactions at positions 2, 3, 4, and 7 to form a variety of dimers, and finally form some high-molecular-weight oligomers. And the oligomers will self-assemble to form multiple cross-linked polymers through the anti disproportionation reaction between catechol and quinone. The surface coating of steel plate was analyzed by Fourier transform infrared spectrometer.

#### 2.2.2. Evaluation of Coating Stability

- The corrosion testing

Polydopamine-coated N80 steel plates prepared under different conditions were obtained by adjusting the pH value of TrIS-HCl buffer solution, the coating temperature and coating time. The corrosion rates of coated N80 steel plates in the produced water of the oil well were measured at a corrosion medium temperature of 80 °C for 7 days. In this way, the best coating preparation conditions were obtained. With reference to Evaluation Method for Behavior of Corrosion Inhibitor for Produced Water of Oil Field SY-T 5273-2000, static weight loss method was adopted to measure the corrosion rate (*r_corr_*_0_) and inhibition rate (*η*). And their formulae are as follows:

(1) $$r_{corr}=\frac{8.76\times 10^{4}\times (m-m_{t})}{S_{t}\cdot t\cdot \rho }$$

where *r_corr_*_0_ is the corrosion rate, mm/a; *m* is the mass of the steel plate before the test, g; *m_t_* is the mass of the steel plate after the test, g; *S_t_* is the total surface area of the steel plate, cm^2^; *ρ* is the density of the steel plate, g/cm^3^; *t* is the test time, h.

(2) $$\eta =\frac{r_{corr0}-r_{corr}}{r_{corr0}}\times 100\%$$

where *η* is the inhibition rate, 100%; *r_corr_* is the corrosion rate, mm/a; *r_corr0_* is the corrosion rate of uncoated steel plate, mm/a.

1. Standard curve of dopamine absorbance

First, 5 mL of dopamine solution with concentrations of 10 μg/mL, 20 μg/mL, 30 μg/mL, 40 μg/mL, 50 μg/mL was added in a 25 mL colorimetric tube, and numbered 1, 2, 3, 4, and 5 respectively. Then, 5 mL NaNO_2_ solution with a concentration of 10 g/L and 12.5 mL HAc-NaAc buffer solution with a pH of 5.9 were added into the tubes. After that, distilled water was added into the tubes until they reached 25 mL, and shaken well for later use. The absorbance of five colorimetric solutions at 302 nm wavelength was measured by ultra-violet and visible spectrophotometer. On this basis, the absorbance-concentration curves were plotted.

2. Test solution preparation and absorbance determination

The optimal coating preparation conditions were selected: the pH value of Tris-HCl buffer is 8.5, dopamine concentration is 5 g/L, preparation temperature is 60 °C, preparation time is 4 h, and 0.222 g/L CuSO_4_ and 0.125 g /L H_2_O_2_ are additives. The coated steel plates were respectively immersed in 100 mL of oil well produced water and gas well produced water (which were filtered twice with a microporous membrane), and acid solution (pH value = 3), and stood at 90 °C for 7 days. After that, 5 mL of immersion solution was taken and putted into a 25 mL colorimetric tube, which was mixed with 5 mL NaNO_2_ solution with a concentration of 10 g/L and 12.5 mL HAc-NaAc buffer solution with a pH value of 5.9. Subsequently, distilled water was added into the tube until it reached 25 mL, and shaken well for later use. An ultraviolet-visible spectrophotometer was used to measure the absorbance of 5 colorimetric tube solutions at a wavelength of 302 nm, so as to obtain the dopamine concentration in the soak solution, and to calculate the dopamine shedding rate on the steel plate during the immersion.

3. Evaluation of coating performance

The influences of conditions such as the corrosive medium temperature, the type of corrosive medium, and the steel plate substrates on the corrosion inhibition of coating were investigated. In addition, the application performance of the dopamine coating was evaluated via scanning electron microscopy and electrochemical tests.

4. Electrochemical test

The electrode material used for electrochemical test was N80 (A3, or J55) steel plate, which was polished with 400#, 800# and 1200# sandpaper successively. It was then washed with distilled water, absolute ethanol and acetone and finally dried, and used as blank sample.

The electrochemical impedance spectra and polarization curves of steel plate samples were measured by CS350 electrochemical workstation. The blank sample and the assembled samples were placed in a special PTFE material electrode sleeve, exposing an area of 1 cm^2^. A three-electrode system was adopted: the reference electrode was saturated calomel electrode (SCE), auxiliary electrode was platinum electrode, and working electrode was N80 (A3, or J55) steel electrode. Throughing Lukin capillary connected with the working electrode. The test solutions included produced water from an oil field, produced water from a gas field, and self-mixed acid solution. The samples were immersed in the test solutions for 30 min, and the potentiodynamic polarization curve and impedance spectrum were measured after the self-corrosion potential stabilized. The potentiodynamic scanning rate was 1.0 mV·s^−1^. The AC impedance was tested at open circuit potential with an AC disturbance amplitude of 5 mV and a scanning frequency range of 0.01–100 khz.

## 3. Results and Discussion

### 3.1. Optimization of Coating Preparation Parameters

Tris-HCl buffer pH optimization (s1); Dopamine concentration optimization (s2); Preparation temperature optimization (s3); Coating time optimization (s4).

### 3.2. Characterization of Coating Composition

**Figure 2:**
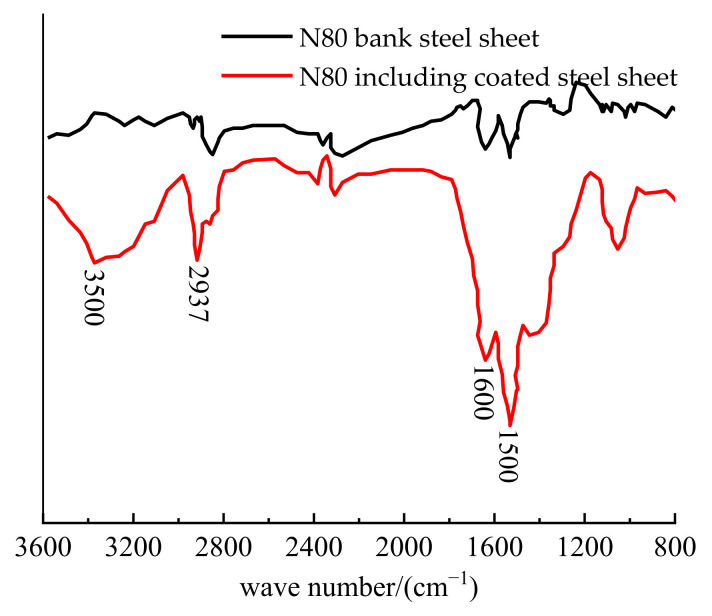
Infrared spectra of polydopamine coating.

Figure 2 shows the infrared spectra of N80 steel plate coated with polydopamine. It can be seen that compared with the blank N80 steel plate, the polydopamine-coated steel plate had a broad peak between 3000–3500 cm^−1^. This was ascribed to the O-H (hydrogen bond) stretching vibration formed between the dopamine molecules or the stretching vibration of the aromatic secondary amine group. The broad peak between 1450–1625 cm^−1^ was the absorption peak of benzene ring skeleton vibration and N-H bending vibration [16,17]. The broad peaks appeared at 1500 cm^−1^ and 1600 cm^−1^ were ascribed to the benzene ring proton, -NH_2_, -NH- and -OH group stretching vibration. The absorption peak at 2937 cm^−1^ was a C-H stretching vibration peak, proving the successful construction of polydopamine coating on the surface of N80 steel.

### 3.3. Stability Test of Polydopamine Coating

The standard curve of absorbance-dopamine concentration was measured by ul-traviolet-visible spectrophotometer, as shown in Figure S5. The absorbance was positively correlated with the concentration of dopamine in the sample solution.

Polydopamine was coated on N80 steel plate under optimum preparation conditions. Then the coated steel plates were respectively immersed in 100 mL of oil well produced water and gas well produced water (which were filtered twice with a microporous membrane), and acid solution (pH = 3), and stood at 90 °C for 7 days. After that, 5 mL of immersion solution was taken out and putted into a 25 mL colorimetric tube, which was mixed with 5 mL NaNO_2_ solution with a concentration of 10 g/L and 12.5 mL HAc-NaAc buffer solution with a pH of 5.9. Subsequently, distilled water was added into the tube until it reached 25 mL, and shaken well for later use. The absorbance of five colorimetric solutions at wavelength of 302 nm was measured by an ultraviolet-visible spectrophotometer, and the concentrations in the soak solution were obtained, as listed in Table 3. It can be seen from Table 3 that the dopamine concentrations in the samples to be tested were extremely low, and the shedding rates were very small. These demonstrate that the polydopamine coating had good stability in oil well produced water, gas well produced water, and acid solution, and was not easy to fall off.

### 3.4. Evaluation of Coating Performance

The coatings were prepared on the surface of steel or electrodes for performance evaluation under the best coating preparation conditions: Tris-HCl buffer solution pH was 8.5, dopamine solution concentration was 5 g/L, coating preparation temperature was 60 °C, coating time as 1 h, and additives were 0.222 g/L CuSO_4_ and 0.125 g/L H_2_O_2_.

#### 3.4.1. Impact of Corrosion Temperature

**Figure 3:**
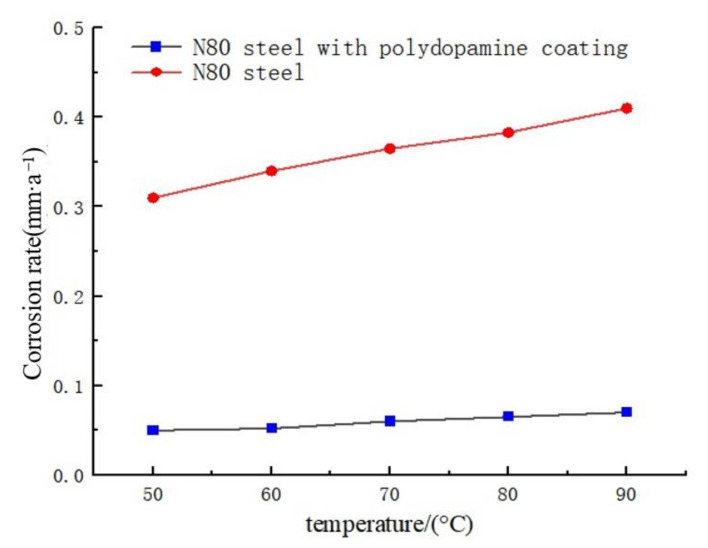
Influence of temperature on the corrosion inhibition of coating.

The coated N80 steel plates prepared under the optimum preparation conditions were placed in the oil well produced water at 50 °C, 60 °C, 70 °C, 80 °C and 90 °C for 7 days. The anti-corrosion properties of coated steel plates were evaluated by static weight loss method, and the results are shown in Figure 3. It can be observed from the figure that the corrosion rate of N80 steel without coating was higher than that of N80 steel with polydopamine coating, proving that the coating could inhibit the corrosion of N80 steel. As the temperature rose, the average corrosion rate gradually increased, and the corresponding corrosion inhibition rates were 84.36%, 84.92%, 81.17%, 81.98% and 81.54%, respectively. It shows that the corrosion inhibition effect of the dopamine coating became worse with the rising temperature, but the corrosion rate was lower than the standard value (0.076 mm·a^−1^) prescribed by the *Evaluation Method for Behavior of Corrosion Inhibitor for Produced Water of Oil Field SY-T 5273-2000*.

**Figure 4:**
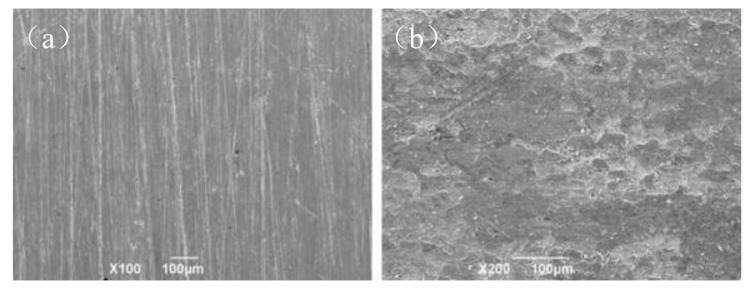
Corrosion inhibition effect of polydopamine coating on N80 steel plate in oil well produced water (90 °C): (**a**) N80 steel sheet with layer; (**b**) N80 steel sheet.

In order to further observe the microcorrosion morphology of N80 steel surface, a corrosion test was performed on N80 steel plates with/without polydopamine coating. The corrosive medium was 90 °C produced water of oil well. Scanning electron microscopy analysis was performed on each corroded steel plate, and the results are shown in Figure 4. It can be seen that the surface of the steel sheet with polydopamine coating after the corrosion test was significantly smoother than that of the blank sample. After corrosion, many pits appeared in the N80 steel plate without coating, indicating that the coating has a good corrosion inhibition effect on N80 steel plate. This may be due to the strong adsorption characteristics of the polydopamine cross-linked layer [18,19].

**Figure 5:**
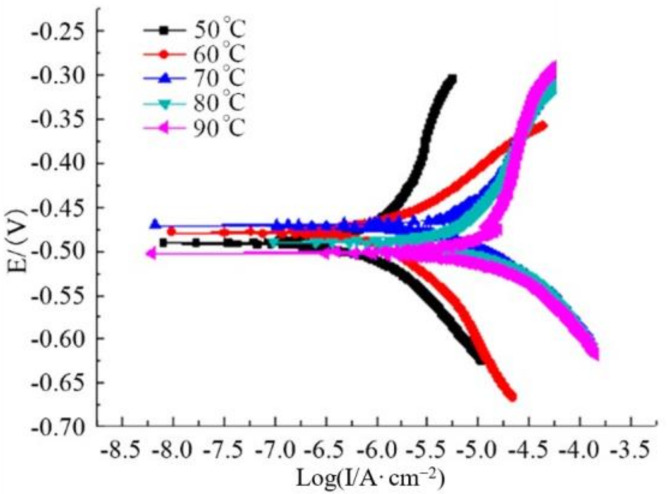
Polarization curves of polydopamine coating at different temperatures.

Figure 5 shows the polarization electrode curve of polydopamine-coated N80 steel plate in oil well produced water. It can be seen that at 50 °C, the anodic polarization curve was the steepest and the corresponding self-corrosion current density was the smallest. This indicates that the corrosion rate of the N80 steel plate was relatively small at this temperature. As the temperature further rose, although the cathode and anode polarization curves shifted to the lower current direction, the anodization curve became gentle and the anode self-corrosion current density increased. It means that the corrosion rate of the metal anode increased with the rising temperature, and the ability of the polydopamine coating to inhibit the anode reaction became worse [20]. Furthermore, the corrosion of anode (N80 steel) tended to aggravate. The results are consistent with those obtained by weight loss method.

**Figure 6:**
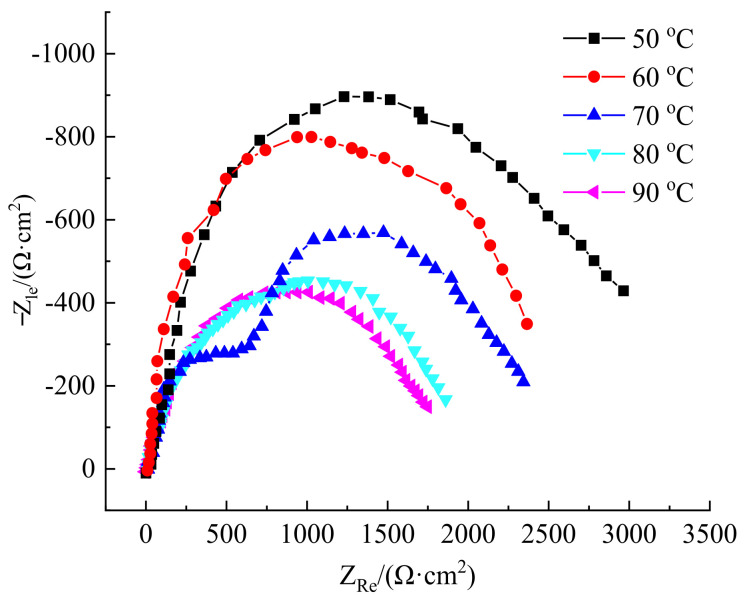
AC impedance diagram of polydopamine-coated N80 steel plate at different temperatures.

Figure 6 shows the polydopamine-coated impedance spectra of N80 steel plates in oil well produced water at different temperatures. It can be seen that capacitive reactance arcs representing charge transfer appeared in impedance spectra at high frequency. As the temperature rose, the capacitive reactance radius of the capacitive reactance arc decreased, indicating a decline in the surface resistance of the metal. It means that the higher the corrosion temperature, the more serious the corrosion of N80 steel plate by oil well produced water. With the increase of temperature, the anti-corrosion ability of polydopamine-coated N80 steel became worse, which is consistent with the results measured by static weight loss and polarization curve.

#### 3.4.2. Impact of Corrosive Media

The polydopamine-coated N80 steel plates prepared under the optimized conditions were placed in the three test soak solutions (oil well produced water, gas well produced water and self-mixed acid solution) of 90 °C for 7 days. The static weight loss method was used to evaluate the anti-corrosion properties of coated steel plate. The corrosion steel plate and corrosion electrode were both N80. The anti-corrosion performance is shown in Table 4. It can be seen that the corrosion rate of N80 steel in the blank test group was greater than that of the polydopamine-coated N80 steel, demonstrating the corrosion inhibition effect of polydopamine coating on N80 steel. Furthermore, polydopamine coated N80 steel plate had different corrosion rates in the three soak solutions, but the difference is insignificant. And the corrosion rates are lower than the standard value (0.076 mm·a^−1^) prescribed by *the Evaluation Method for Behavior of Corrosion Inhibitor for Produced Water of Oil Field SY-T 5273-2000*. It is therefore can be concluded that the polydopamine coating has excellent anti-corrosion ability in oil well produced water, gas well produced water, and acid solution [21].

**Figure 7:**
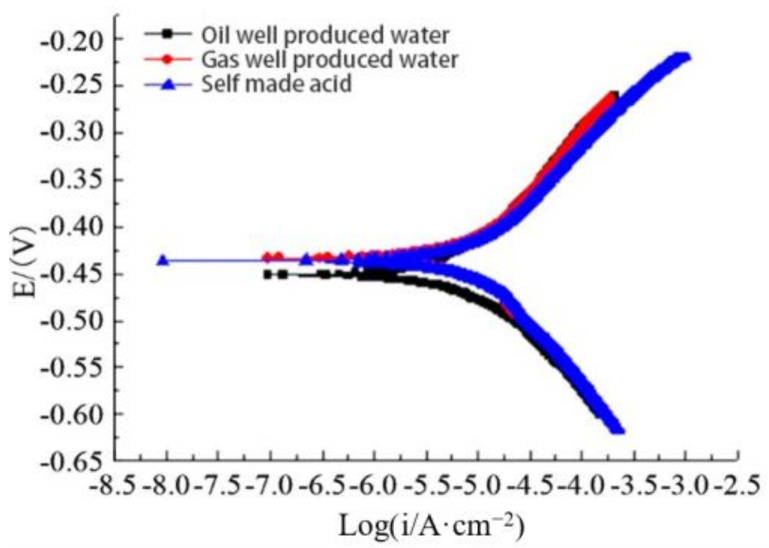
Polarization curves of polydopamine coating in corrosive media.

Figure 7 shows the polarization curve of polydopamine-coated N80 steel plate in oil well produced water, gas well produced water, and acid solution. It can be observed that the polarization curves of the electrode in the three solutions had no obvious difference. In addition, the variation trends of the cathode and anode polarization curves were close, so were the corresponding current density ranges. These indicate that the dopamine coating had similar corrosion inhibition ability in various corrosive media, which is consistent with the results by the static weight loss method.

**Figure 8:**
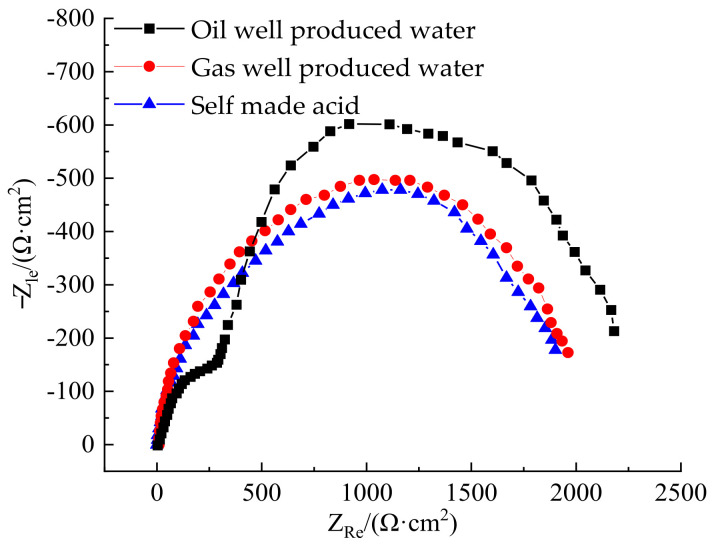
AC impedance diagram of polydopamine-coated N80 steel plate in corrosive media.

Figure 8 shows the impedance spectra of polydopamine-coated N80 steel plate in different corrosive media. As can be seen from Figure 8, the impedance spectra of the coated N80 steel plate in the three media of oil well produced water, gas well produced water, and acid solution were relatively close, so were the arc shapes and radii of capacitive reactance. These prove that the corrosion inhibition properties of polydopamine-coated N80 steel plate in the three corrosive media were similar, consistent with the results measured by the static weight loss and the polarization curve.

#### 3.4.3. The influence of Test Material

The polydopamine-coated N80, A3 and J55 steel plates prepared under the optimum conditions were placed in 90 °C oil well produced water for 7 days. The static weight loss method was used to evaluate the corrosion of the coated steel plate, and the results are shown in Table 5. The corrosion rate of uncoated plates was greater than that of polydopamine-coated ones, so dopamine coating had obvious corrosion inhibition effects on the three kinds of steel materials. Moreover, the corrosion rates of polydopamine-coated N80, A3, and J55 steel plates were different, but their corrosion rates and inhibition rates were close. And the corrosion rates were lower than the standard value in the Evaluation Method for Behavior of Corrosion Inhibitor for Produced Water of Oil Field SY-T 5273-2000, indicating polydopamine coating had good anti-corrosion effect for N80, A3 and J55 steels.

**Figure 9:**
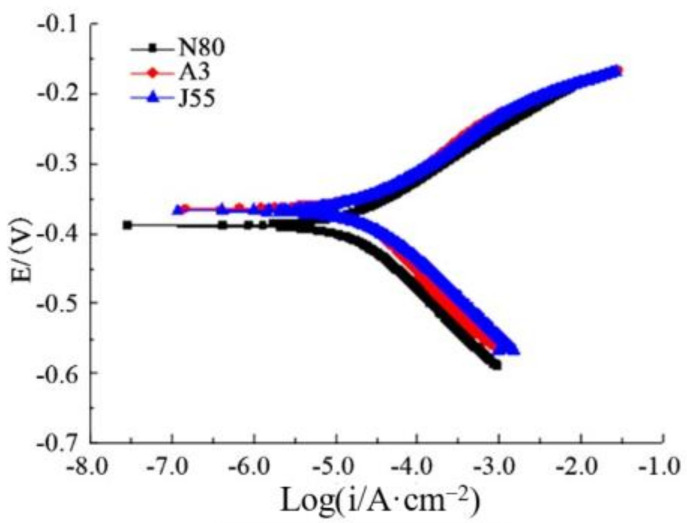
Polarization curves of different steels coated with polydopamine.

Figure 9 presents the polarization curves of three kinds of polydopamine-coated steels in oil well produced water. The polarization curves of the three kinds of steel plates showed no obvious difference, the change trends of the cathode and anode polarization curves were similar, and the current density ranges corresponding to the polarization curves were close. These mean that the coating had similar anti-corrosion ability for N80, A3 and J55 steel plates, close to the results obtained by the static weight loss method [22].

**Figure 10:**
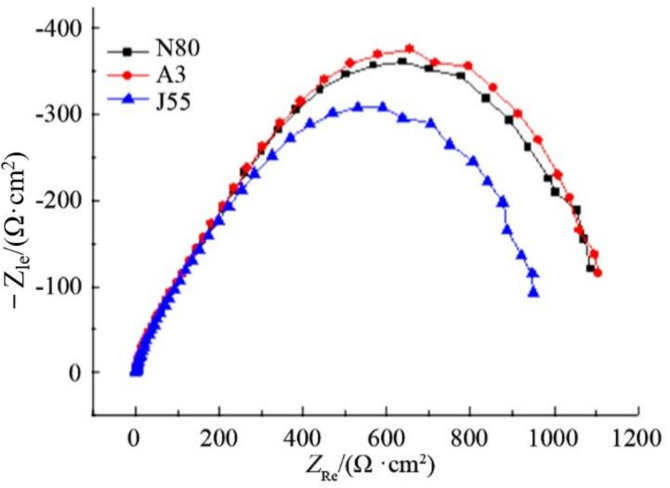
AC impedance diagram of different steel plates with polydopamine coating.

Figure 10 shows impedance spectra of different polydopamine-coated steel plates in oil well produced water. It can be seen that there is no obvious difference in impedance spectra of the steel plates. The arc shapes and radii of capacitive reactance were close, indicating that the coating had similar corrosion inhibition effect on N80, A3 and J55 steel plates in oil well produced water. The results agree with those obtained by weight loss method and polarization curve method.

## 4. Conclusions

In summary, the evidence from this study intimates that through the self-polymerization and assembly of dopamine, a corrosion-inhibiting coating is built on the N80 steel, which has an excellent corrosion-inhibiting effect. The optimal conditions for preparing polydopamine coatings are as follows: the pH of the Tris-HCl buffer solution was 8.5, the dopamine concentration was 5 g/L, the coating temperature was 60 °C, and the coating time was 1 h. The polydopamine coating has a low shedding rate after being immersed in 90 °C oil well produced water, gas well produced water and self-mixed acid solution for 7 days, which indicates that the polydopamine coating has good stability. Although the corrosion rate of polydopamine-coated N80 steel plate in the produced water of oil wells increases with the increase of temperature, they are all lower than the industry standard of 0.076 mm·a^−1^. In addition, the corrosion rates of N80, A3 and J55 steel plates coated with polydopamine coatings in the produced water of oil wells at 90 °C are very similar, indicating that they have good corrosion inhibition to other steel plates. The electrochemical test results also prove this conclusion. In general, the results accumulated from this research will open up a new way to develop more types and high-efficiency anti-corrosion coatings, and show huge industrial application potential.

## Figures and Tables

**Table 1:** Composition of produced water.

Composition	Oil Well Produced Water (Changqing Oilfield)	Gas Well Produced Water (Yulin Gas Field)
Ca^2+^/(mg·L^−1^)	1526.36	9028.02
Na^+^/(mg·L^−1^)	26,576.09	23,782.47
Mg^2+^/(mg·L^−1^)	383.91	302.32
Cation content/(mg·L^−1^)	28,486.36	33,150.51
Cl^-^/(mg·L^−1^)	42,154.48	52,959.11
S^2-^/(mg·L^−1^)	0.6	11.86
SO_4_^2-^/(mg·L^−1^)	2228.79	338.13

**Table 2:** Composition of experimental steel (mass fraction/%).

Material	C	Si	Mn	P	S	Cr	Mo	Ni
N80	0.240	0.220	1.190	0.013	0.003	0.036	0.021	0.028
A3	0.140–0.220	>0.300	0.300–0.650	>0.045	>0.005	\	\	\
J55	0.340	0.200	1.250	0.020	0.015	0.150	\	0.200

**Table 3:** Stability of polydopamine coating.

Soak Solution	Absorbance	Dopamine Concentration/wt%	Shedding Rate/%	Average Shedding Rate/%
Oil well produced water	0.028	3.2184	1.95	1.88
	0.025	2.8736	1.72	
	0.026	2.9885	1.88	
Gas well produced water	0.029	3.3333	1.96	2.01
	0.030	3.4483	2.08	
	0.028	3.2184	1.99	
Acid solution	0.032	3.6782	2.15	2.20
	0.032	3.6782	2.23	
	0.033	3.7931	2.22	

**Table 4:** Influence of corrosive media on the corrosion inhibition of polydopamine coating.

Soak Solution	Steel Plate	Corrosion Rate/mm·a^−1^	Inhibition Rate/%
Oil well produced water	N80	0.3618	81.69
	Polydopamine-coated N80	0.0554	
Gas well produced water	N80	0.3694	86.14
	Polydopamine-coated N80	0.0512	
Acid solution	N80	0.3983	85.16
	Polydopamine-coated N80	0.0591	

**Table 5:** Corrosion inhibition effect of polydopamine coating on different steels.

Steel	Is It Coated with Polydopamine	Corrosion Rate/mm·a^−1^	Inhibition Rate/%
N80	No	0.3894	86.11
	Yes	0.0541	
A3	No	0.3626	86.27
	Yes	0.0498	
J55	No	0.3362	86.47
	Yes	0.0455	

## Data Availability

The data that support the findings of this study are available from the corresponding author upon reasonable request.

## Supplementary Materials

The following supporting information can be downloaded at: https://www.mdpi.com/article/10.3390/polym14040794/s1, Figure S1: Influence of Tris-HCl buffer solution’s pH value on the corrosion inhibition of coating. s2, Figure S2. Influence of dopamine concentration on the corrosion inhibition of coating. s3, Figure S3: Influence of preparation temperature on the corrosion inhibition of coating. s4, Figure S4. Influence of coating time on the corrosion inhibition of coating. Figure S5: Standard curve of dopamine concentration-absorbance. In addition, references [23,24,25,26,27,28,29] are cited in the supplementary materials.
