# Supplementary material for: Corrosion Inhibition Coating Based on the Self-Assembled Polydopamine Films and Its Anti-Corrosion Properties
Source: Polymers (Basel). 2022 Feb 18;14(4):794. doi: 10.3390/polym14040794 (PMC8875011; doi:10.3390/polym14040794)
Supplement: Supplementary file 1 [file polymers-14-00794-s001.zip › polymers-1494230-supplementary.pdf]

# Supporting Materials

## Corrosion Inhibition Coating Based on the Self- Assembled Polydopamine Films and Its Anti-Corrosion Properties

**Shanjian Li [1,*], Changwang Guo [1], Xin Wang [2], Chong Guan [1,2] and Gang Chen [1,3]**

1 Shaanxi Province Key Laboratory of Environmental Pollution Control and Reservoir Protection Technology of Oilfields, College of Chemistry and Chemical Engineering, Xi'an Shiyou University, Xi'an 710065, China

2. No. 3 Gas Production Plant of Yanchang Gas Field of Shaanxi Yanchang Petroleum Co., Ltd, Yan'an 716000, China

3 State Key Laboratory of Petroleum Pollution Control, Xi'an Shiyou University, Xi'an 710065, China

* Correspondence: lishanjian @xsyu.edu.cn; Tel.: +86-029-88382692

Optimization of coating preparation parameters

**S1.** Tris-HCl buffer pH optimization

The dopamine concentration was 5 g/L, the preparation temperature was 60 °C, the coating time was 1 h, and the additives were 0.222 g/L$CuSO_4$ and 0.125 g/L $H_2O_2$. The corrosion rates of polydopamine-coated N80 steel plates prepared when the pH value of Tris-HCl buffer solution was 7.5, 8.0, 8.5, 9.0 and 9.5 respectively were measured in the oil well produced water. The results are shown in Figure S1. When the pH was between 7.5 and 8.5, the corrosion rate decreased with the increasing pH; when the pH ranged from 8.5 to 9.5, it increased with the rising pH. It indicates that the dopamine coating had the best corrosion inhibition effect when the pH of buffer was 8.5. This is because the oxidation reaction of dopamine is the first step of the self-polymerization-assembly reaction of dopamine, and it needs to take place in an alkaline environment. Moreover, high alkaline will inhibit the reaction, so a moderate alkaline environment (pH: 8.5) is the optimal [1-3].

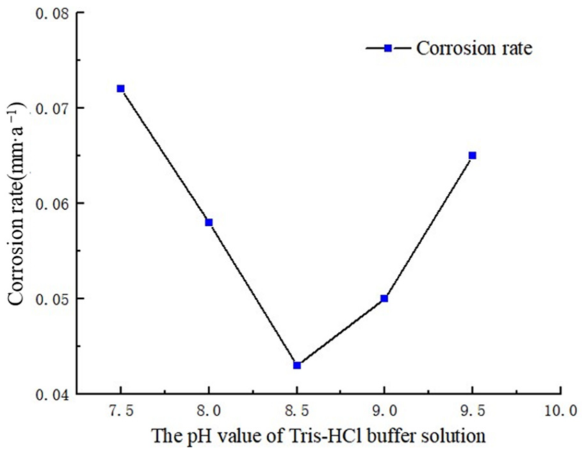


**Figure S1.** Influence of Tris-HCl buffer solution's pH value on the corrosion inhibition of coating.

**S2** Dopamine concentration optimization

The preparation temperature was 60 °C, the pH of the Tris-HCl buffer was 8.5, the coating time was 1 h, and the additives were 0.222 g/L $CuSO_4$ and 0.125 g/L $H_2O_2$. The corrosion rates of polydopamine-coated N80 steel plates, prepared at dopamine concentrations of 1 g/L, 2 g/L, 5 g/L, 7 g/L and 10 g/L, in the produced water of oil well were measured. The results are presented in Figure S2. When the dopamine concentration was 1 g/L, 2 g/L, 5 g/L, 7 g/L, and 10 g/L, the corresponding corrosion rates 0.0987 mm/a, 0.0638 mm/a, 0.0535 mm/a, 0.0499 mm/a, 0.0481 mm/a, respectively. The corrosion rate decreased with the rising dopamine concentration, indicating that the coating was more stable with the increase of dopamine concentration. When the dopamine concentration was above 5 g/L, the corrosion rate showed little change with the dopamine concentration. This is consistent with the results from reported research [4-5]. Ball et al. found that as the concentration of dopamine changed from 0.1 to 5 g/L, the thickness of the polydopamine film showed a gradually increasing trend. And when the concentration of dopamine was 5 g/L, the film thickness could reach 81 nm [6]. However, the thickness of the polydopamine film was limited by the dissolved oxygen in water, so the corrosion effect did not change much as the concentration dopamine increased.

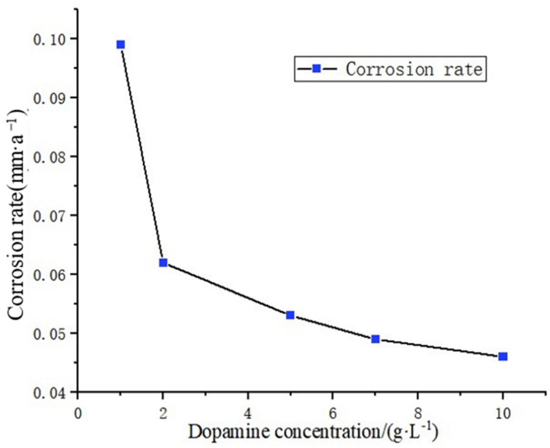


**Figure S2**. Influence of dopamine concentration on the corrosion inhibition of coating.

**S3** Preparation temperature optimization

During the coating, the dopamine concentration was set to 5 g/L, the pH of Tris-HCl buffer to 8.5, the coating time to 1 h, the additives to be 0.222 g/L $CuSO_4$ and 0.125 g/L $H_2O_2$. The corrosion rates of polydopamine-coated N80 steel plates prepared at 20 °C, 40 °C, 60 °C and 80 °C in oil well produced water were measured. The results are shown in Figure S3. It can be observed that as the corrosion rate declined with the rising coating temperature, indicating that the higher the preparation temperature, the better the corrosion inhibition performance. But when the temperature rose from 60 °C to 80 °C, the corrosion rate did not change much. Thus, 60 °C was selected as the best coating preparation temperature. As reported by Shin et al [7], under vigorous stirring, an increase in the reaction temperature would increase the deposition rate of polydopamine, thereby optimizing the polydopamine coating and reducing the corrosion rate.

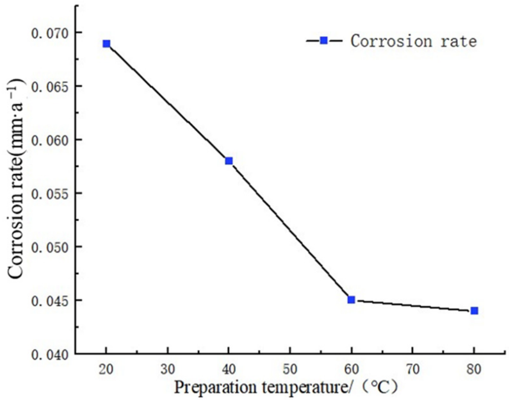


**Figure S3.** Influence of preparation temperature on the corrosion inhibition of coating.

**S4** Coating time optimization

During the coating, the dopamine concentration was set to 5 g/L, the pH of Tris-HCl buffer to 8.5, the preparation temperature to 60 °C, and the additives to be 0.222 g/L $CuSO_4$ and 0.125 g/L $H_2O_2$. The corrosion rates of N80 steel plates coated with polydopamine in the oil well produced water were determined when the coating time was 1 h, 2 h, 3 h and 4 h, respectively. The results are presented in Figure S4. As the coating time increases, the corrosion rate decreases slightly. This indicates that the increase in time will enhance the corrosion inhibition of the coating, but the improvement is not obvious. Therefore, 1 h was determined as the best coating time. The result further confirms that dopamine monomers require at least 1 h of deposition time to form a dense film without pores, as reported in the study. One hour on, the time had little effect on the corrosion effect of the film as it further increased.

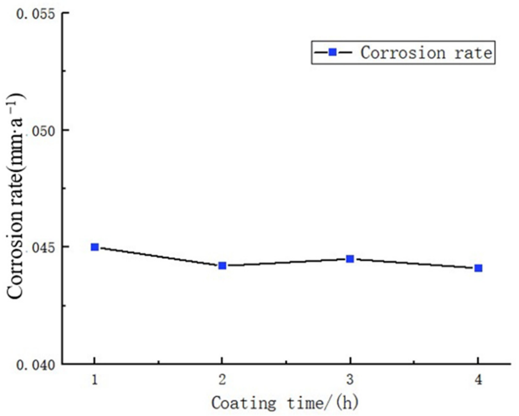


**Figure S4.** Influence of coating time on the corrosion inhibition of coating.

**S5.** The standard curve of absorbance-dopamine concentration

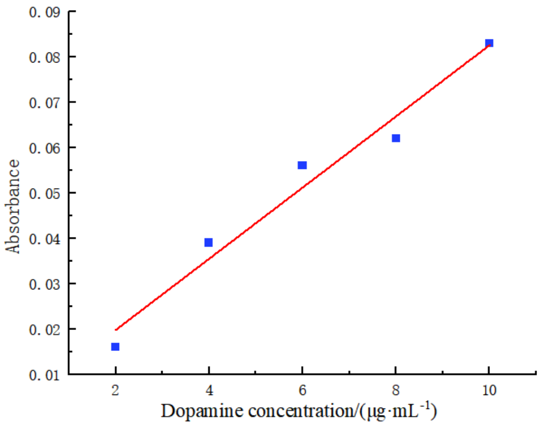


**Figure S5**. Standard curve of dopamine concentration-absorbance.

## References


1. Postma A.; Yan Y.; Wang Y.J. Self-polymerization of dopamine as a versatile and robust technique to prepare polymer capsules. *Chem. Mater.* **2009**, *21*, 3042-3044.
2. Ye Q.; Zhou F.; Liu W.M. Bioinspired catecholic chemistry for surface modification. *Chemical Society Reviews*. **2011**, *40*, 4244-4258.
3. Qu J.F.; Wang J.Q.; Liu S. Microtribological and electrochemical corrosion behaviors of polydopamine coating on APTS-SAM modified Si substrate. *Appl. Surf. Sci.* **2009**, *256*, 894-899.
4. Zhang L, Shi J, Jiang Z, Jiang Y.; Meng R.; Zhu Y.; Liang Y.; Zheng Y. Facile preparation of robust microcapsules by manipulating metal-coordination interaction between biomineral layer and bioadhesive layer[J]. *ACS Appl. Mater. Interfaces*, **2011**, *3*, 597-605.
5. Batul R.; Tamanna T.; Khaliq A.; Yu A. Recent progress in the biomedical applications of polydopamine nanostructures. *Biomater. Sci.* **2017**, *5*, 1204-1229.
6. Yang F.; Li X.; Dai Z. Liu T.; Zheng W.; Zhao H. Corrosion inhibition of polydopamine nanoparticles on mild steel in hydrochloric acid solution. *Int. J. Electrochem. Sci*. **2017**, *12*, 7469-7480.
7. Lee H.; Scherer N.F.; Messersmith. Single-molecule mechanics of mussel adhesion. *Proc. Natl. Acad. Sci.* **2006**, *103*, 12999-13003.
